# Pericytes in mouse heart

**DOI:** 10.3389/fphys.2025.1631407

**Published:** 2025-07-30

**Authors:** Guiling Zhao, W. Jonathan Lederer

**Affiliations:** ^1^ Laboratory of Molecular Cardiology, Center for Biomedical Engineering and Technology, University of Maryland School of Medicine, Baltimore, MD, United States; ^2^ Department of Pharmacology and Physiology, University of Maryland School of Medicine, Baltimore, MD, United States

**Keywords:** heart, pericyte, microcirculation, capillary, confocal imaging

## Abstract

Pericytes are cells associated primarily with capillaries and are thought to play an important role in the regulation of blood flow. They are often referred to as “mural” cells because they are so frequently found on the exterior walls of small vessels - particularly the capillaries. In heart, high-resolution real-time observations and measurements of pericyte function under physiological conditions are challenging to obtain because of vascular motion, tissue depth and vigorous functional movement. For these reasons, the heart may be one of the most difficult tissues in which to examine pericyte function. Recently, we introduced a perfused papillary muscle preparation (the Z-Prep) that allows us to observe coronary arteries, arterioles, venules, capillaries and myocytes in real time at physiological temperature and pressure while also imaging pericytes. Here we present an initial study intended to visualize and characterize quantitatively cardiac pericytes in heart at physiological pressure and temperature conditions. Vascular anatomy was imaged using a z-stack protocol with a rapidly spinning disk confocal microscope. Here the anatomical organization of the pericytes is shown at high resolution with respect to the microcirculation components and cardiac myocytes. The surprising findings include the high abundance of pericytes in native tissue, the extent of their spread on the capillaries themselves, and the existence of major pericyte extensions that travel intimately along the surface of neighboring ventricular myocytes and attach to capillaries on the distant side. These extensions arise from a capillary-based pericyte location and normally end on another capillary endothelial surface and we have named them “bridging” pericytes. Taken together this anatomical organization suggests that the pericytes provide signaling, communication and contractile services to important cellular components of the heart. There is also a suggestion that pericytes in heart are unusually fragile since they suffer an extremely high degree of loss during cellular isolation procedures. However, our investigation of the organization argues against this fragility because of the durability of the dynamic pericyte organization and function despite the stress and brutality of the contracting heart. The work presented here lays the foundation for critical functional studies of pericytes in heart in both health and disease.

## Introduction

Within the complex landscape of the microvasculature in heart, understanding the capillary functional organization is of utmost importance. Here we attempt to extend our understanding of the roles played by pericytes. It has been hypothesized, for example, that the rapid and accurate control of local blood flow by small vessels in the heart is critical for healthy normal heart function, that it is driven by the metabolic state of the contractile tissue and that the capillary endothelium communicates this signal to contractile elements that include the pericytes and the vascular smooth muscle cells on arterioles (Kovac et al., 2020). This process has been dubbed “electro-metabolic signaling (EMS)” (Zhao et al., 2020a; Longden and Lederer, 2024; Longden et al., 2023). EMS mechanisms primarily contain 3 cell types (cardiac myocyte, pericyte, and local vascular smooth muscle cells) and that each is connected to the others via local endothelial cells (Zhao et al., 2020a; Jackson, 2022; Ledoux et al., 2008). However, many aspects of EMS remain unclear, especially the function and involvement of the pericytes, due largely to the inherent complexities of studying these components in the beating heart. The relentless motion and rhythmic contractions of the heart make *in vivo* and *ex vivo* investigations exceptionally demanding [see reviews (Kalia, 2023; Vigneshwaran et al., 1994; Kavanagh and Kalia, 2019)]. Furthermore, the pursuit of real-time imaging with observations of these structures within the dynamic cardiac environment pushes the boundaries of current imaging technological capabilities. Consequently, most of our current knowledge about coronary microvessels and their pericytes comes from fixed, static heart tissues (Seidel et al., 2016; Bassingthwaighte et al., 1974; O'Farrell et al., 2017; Higuchi et al., 2000). A lot of elegant very recent work has already revealed the sophisticated structures of the heart microcirculation and the local architecture of the mural cells (e.g., pericytes, vascular smooth muscle cells). Such recent work has advanced our knowledge of cardiovascular microcirculation biology (Higuchi et al., 2000; Kassab et al., 1993; Kassab and Fung, 1994). However, the process of tissue fixation can cause damage, deformation, and loss of cells and other components, leading to possible inaccurate observations and data interpretation. Additionally, one-time snapshot images cannot capture the dynamic changes that occur under diverse conditions including those that are stressful and challenging. Certainly, our understanding based on past studies of physiological and pathological changes in the microcirculation (based primarily on fixed tissue) should be verified. Thus, efforts to image live tissues *in vivo* and *ex vivo* have also been ongoing and are important (Kalia, 2023; Qin et al., 2016; Li et al., 2012; Lee et al., 2012; Vinegoni et al., 2012; Matsuura et al., 2018). Significant advances in live-tissue imaging have been achieved in brain research in recent years (Berthiaume et al., 2018; Hartmann et al., 2015; Ornelas et al., 2021). However, less progress has been made in heart research due to the non-stop beating of the heart and the difficulty of performing surgery to allow long-term high speed imaging of the dynamics of its vasculature.

To investigate active structural and functional changes in the heart, we recently developed a mouse papillary muscle preparation (Z-Prep) that allows us to pressurize the blood vessels and perfuse the tissue (Zhao et al., 2020a; Longden et al., 2023; Zhao et al., 2020b). This preparation enables high-resolution three-dimensional visualization of the microvascular bed. Using the Z-Prep, we successfully captured the microvascular diameter change in the presence of vasodilators and vasoconstrictors (Zhao et al., 2020a; Zhao et al., 2020b). On the other hand, confusion remains regarding the identification of different mural cells and their respective functions in the heart. A more detailed characterization of the morphology of different mural cell types and the microvascular territories they occupy is needed to aid in the interpretation of the functional behavior of heart pericytes, both physiologically and pathologically. In this study, we aim to describe and advance our understanding of the structures of the “real” microvascular beds and illustrate the architecture of the surrounding components with an emphasis on pericytes. Using NG2-DsRed and PDGFRβ-tdTomato transgenic mice, we have characterized and categorized pericytes according to their locations, morphologies, and the presence of smooth muscle actin. Although the classification of pericytes has historically been challenging due to their phenotypic plasticity and the overlap of marker expression with other cell types, our efforts have provided important clarity. Finally, the function of pericytes in the regulation of microvascular blood flow is discussed based on the results presented here.

## Methods

### Preparation of arterially perfused mouse right ventricle papillary muscle-Z-Prep

**FIGURE 1:**
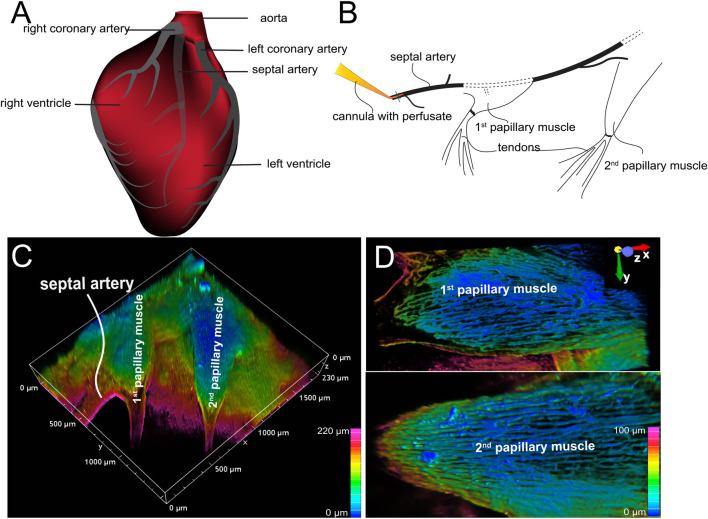
Arterially perfused mouse right ventricle papillary muscle. **(A)** Cartoon shows the origin and course of coronary arteries. Note: The septal artery normally originates from the right coronary artery. **(B)** Diagram shows the location of cannula and the papillary muscles. **(C)** 3D depth-encoded vascular imaging of a Z-prep using WGA conjugated with Alexa Fluor 488 as fluorescent marker of the vasculature over a depth of 100 microns. **(D)** Zoomed-in images from **(C)** with first (top) and second papillary muscle (bottom). Depth encoding over 100 microns.

The pressurized and arterially perfused mouse right ventricle papillary muscle (Z-Prep) was prepared as described previously (Zhao et al., 2020a; Zhao et al., 2020b) (Figure 1). In brief, the hearts of a total of 36 mice (11 wild type, 24 NG2-DsRed and 1 PDGFRβ-tdTomato) were removed under anesthesia and placed into ice-cold Tyrode’s solution (in mM: 140 NaCl, 10 HEPES, 0.5 MgCl_2_, 0.33 NaHPO_4_, 5.5 Glucose, 1.8 CaCl_2_, and 5 KCl (pH 7.4 with NaOH)) containing 30 mM BDM (2,3-Butanedione monoxime). After cleaning the heart of connective tissues, it was transferred into a pre-chilled chamber that had been coated with PDMS (polydimethylsiloxane) and filled with Tyrode’s solution containing BDM (30 mM). Then the right ventricle was cut open carefully and the right ventricular free wall was removed. The septal artery was exposed (see cartoon picture Figure 1B). A nylon thread (30 µm in diameter, Living Systems Instrumentation, Burlington, VT, USA) was placed under the sepal artery and a loose knot was tied. The left ventricular free wall was removed, and the sample was transferred into the PDMS-coated experimental chamber (filled with Tyrode’s solution containing BDM) in which the cannula was positioned. The septal artery was then cannulated, and small pins were used to secure the papillary muscle to the chamber floor so that the microscope objective had a clear view of the vasculature. Then the cannula was connected to a pressure/flow control pump or a gravity column to allow pressurized perfusion into the vascular lumen. The bath was superfused with physiological saline solution (PSS, in mM: 112 NaCl, 5 KCl, 1.2 MgSO_4_, 1.2 NaH_2_PO_4_, 24 NaHCO_3_, 10 glucose, 1.8 CaCl_2_, bubbled with 5% CO_2_) at ∼2–3 mL/min at 35°C–37°C. The perfusion pressure was monitored using a pressure transducer as needed and as previously reported (Zhao et al., 2020a; Zhao et al., 2020b). The arterially perfused septum and papillary muscles were studied at physiological pressure, ranging from 40 mmHg to 80 mmHg. The perfusate temperature was controlled at room temperature or physiological temperature (∼35°C–37°C, for functional investigations) using an inline heater. Unless specified otherwise, BDM (30 mM) was included in the perfusates to minimize the motion of the tissue.

### Dye loading, imaging and photo bleaching

After ∼30 min of stabilization, Alexa Fluor-488 or 633 conjugated wheat germ agglutinin (WGA, 20 μg/mL) or Isolectin B4 (IB4) was loaded into the arterial lumen perfusate for ∼30 min followed by a complete wash. All live images were acquired using an upright Nikon A1R microscope. The papillary muscle (or septum) was first located visually and imaged using a ×4 objective. Subsequently, a high-power objective (×40 W DIC M N2) was used to image vessels and pericytes. For structural measurements, high resolution imaging was performed using either a z-stack spinning disk (2048 × 1,024 pixel, 0.14–0.23 µm/pixel) or point scan confocal (512 × 512 pixel, 0.41–0.62 µm/pixel) with a z-step interval of 0.75–1 μm. Lasers at 488, 561, or 647 nm were used to excite DsRed, Fluor-488 and Fluor-633, respectively. Fluorescence was collected using 525/50, 595/50, and >700 nm emission filters, respectively. When biochemical agents such as endothelin-1 (ET-1, 5 or 10 nM) and BDM (30 mM) were applied, time-series imaging using XY or XYZ modes was conducted. Refocusing was often required during imaging due to tissue motion or changes in vessel diameter. For photo bleaching experiments targeting DsRed, a 5 × 5 μm area on the pericyte soma was selected. 561 and 405 nm lasers at 100% power were applied for 20 s to 2 min.

### Whole mount staining and immunostaining procedure

WT or NG2-DsRed mouse hearts were cannulated and perfused using the Langendorff perfusion system with Ca^2+^-free Tyrode’s solution at room temperature. IB4 (10 μg/mL) or WGA (20 μg/mL), Mitotracker (5 μg/mL), and Hoechst or DAPI (10 μM) were loaded for 30 min, followed by a 10-min wash. Subsequently, 4% paraformaldehyde (in PBS) was perfused through the vasculature for 15 min to fix the heart, and this perfusion was followed by a 5-min wash using PBS. The heart was then removed from the Langendorff perfusion rig, and imaging was performed on either the ventricles or the papillary muscle. For whole mount immunostaining, after dye loading, wild-type hearts were permeabilized using 0.1% or 0.2% Triton X-100 (in PBS) for 15 min. The heart was removed from the Langendorff perfusion rig, and the right ventricular papillary muscle was dissected for whole-mount immunostaining. The tissue was incubated with primary antibodies—anti-NG2 (MAB5384, 1:100; Millipore Sigma-Aldrich) and anti-SMC-actin (Ab5694, Abcam)—for over two nights (∼36 h) at 4°C in a blocking solution (5% goat serum and 0.1% or 0.2% Triton X-100 in PBS), followed by a 2-h wash with PBS. The tissues were then incubated with an Alexa Fluor-conjugated secondary antibody (1:200 to 1:3,000) for 4–6 h at room temperature, followed by an overnight wash with PBS at 4°C. The tissues were then either mounted on a Sylgard-coated chamber using a fine needle or glued onto a coverslip and imaged using upright confocal microscopy (Nikon A1R).

### Transmission electron microscopy (TEM) myograph

Transcardiac perfusion was used to fix the mouse heart. In brief, under deep anesthesia with isoflurane, the mouse chest was opened and the beating heart was exposed. A 21-gauge perfusion needle was passed through the left ventricle into the ascending aorta. A hemostat is used to clamp the heart to secure the needle and prevent leakage. A small incision was made in the animal’s right atrium using delicate scissors. After verification that there are no bubbles in the infusion fluid lines, transcardiac perfusion was started with phosphate-buffered saline (PBS) until the fluid exiting the right atrium is entirely clear. Switch from PBS to fixative containing 2% paraformaldehyde and 2.5% glutaraldehyde in 0.1 M sodium cacodylate buffer (pH 7.4) and perfused 20–30 mL of fixative at ∼80 mmHg using gravity-fed system. The heart was then removed and placed in the fixative overnight. The right ventricular papillary muscle was subsequently dissected and placed into a buffer containing 1% osmium tetroxide in 0.1 M sodium cacodylate (provided by the core facility). The sample was then submitted to the Electron Microscopy Core Imaging Facility (EMCIF) at the University of Maryland, Baltimore, for further processing. Briefly, the samples were stained with 1% tannic acid, followed by serial dehydration in ethanol. They were embedded in EmBed812 resin and polymerized at 60°C for 48 h. Ultrathin sections (70 nm) were mounted on copper grids and imaged using a Thermo Fisher Tecnai T12 TEM at 80 kV. Images were captured with a 16 MP AMT XR-60B CMOS camera.

### Image processing and data analysis

When necessary, images were first processed using Nikon Imaging Software (NIS Elements, version 5.21.03). Specifically, image alignment and de-noising were performed automatically using “ND Processing-Align Current Document” and “NIS.ai” functions. The processed images were then further processed and analyzed using either the General Analysis 3 (GA3) module in NIS or Imaris (10.0.1, Oxford Instruments). Briefly, pericytes and the nearest distances between them were identified and quantified using 3D Spot Detection. Bright spots were defined as structures with a typical diameter 11–15 μm with contrast adjusted accordingly. The detection, counting, and the nearest-distance measurements of these bright spots were conducted using a customized NIS GA3 workflow. Volumes of imaged tissue, capillaries and pericytes were measured via 3D thresholding using GA3, ensuring all cellular processes and capillaries were included in the thresholded images. Volume ratios were calculated relative to the total imaged tissue volume. Similarly, myocyte volumes were measured from cells that were injected with a fluorescence marker Lucifer Yellow (10 μg/mL in 1 M LiCl).

Capillary segment length was measured semi-automatically using Imaris. Confocal image files were first converted into.ims format using ImarisFileConverter, followed by 3D volume reconstruction. Segment measurements began with selecting branch points using “Measurement Points” tool. For short and straight segments, the “Pairs” mode in Line Mode was used; otherwise, the “Polygon” mode was applied. Vessel diameter was measured in a similar manner. After testing serval approaches, we determined this to be the most accurate method for segment length and diameter quantification. Capillary local narrowing was defined as a discrete and localized reduction in capillary diameter that is distinguishable from adjacent capillary segments. All the data was presented as Mean ± SEM. Unless otherwise stated, statistical analysis was performed using two sampled t-test in Origin 2018, with significance value set at P < 0.05.

## Results

### Capillary network geometry

Typically, the septal artery originates from the right coronary artery (illustrated in Figure 1A). However, in some instances, it can arise from the left coronary artery (Yoldas et al., 2010). At lower magnifications (using a ×4 objective lens), two or more papillary muscles within the same field of view could be observed, as shown in Figures 1B,C (Zhao et al., 2020a; Zhao et al., 2020b). These papillary muscles exhibited varying shapes: the first one appeared relatively round; the second elongated, and the third was also elongated but smaller (Figures 1B–D). Figure 1C presents a typical Z-Prep, showcasing the septum and mouse papillary muscles. Due to its proximity to the cannula, the first papillary muscle is a feasible choice for imaging and data collection. However, the second papillary muscle is often preferred (Figures 1B–D) because it is comparatively thinner, smaller, flatter, and better perfused. These characteristics facilitate more straightforward and consistent imaging (Figure 1D). The third papillary muscle which is located deeper was more challenging to keep in focus and thus less frequently studied. On occasion, well-perfused areas of the septum were also imaged.

### The vascular organization

**FIGURE 2:**
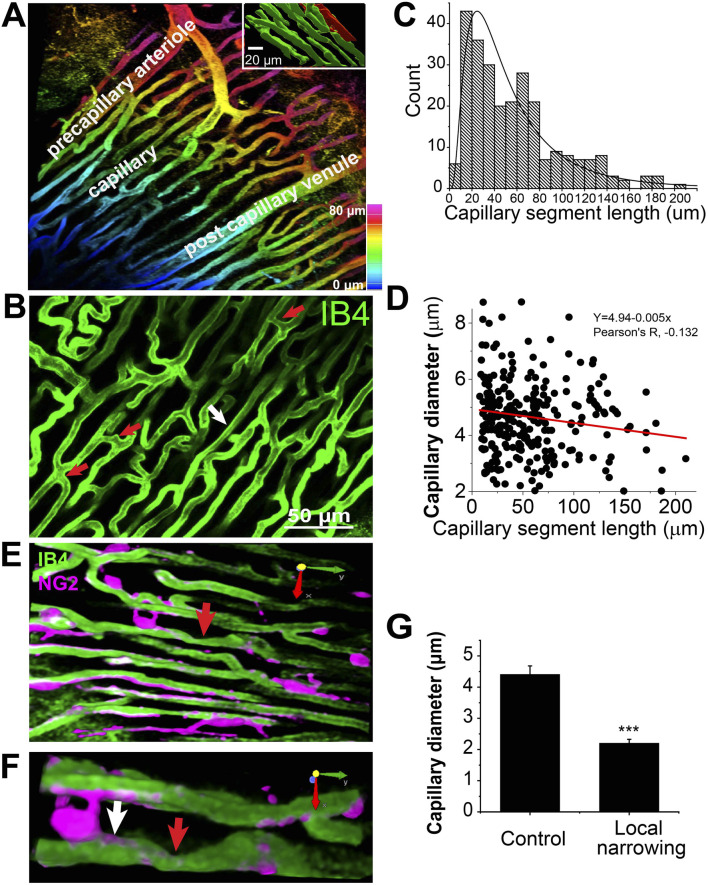
Capillary network geometry and the size in Mouse Heart (Z-Prep). **(A)** A 3D image with colored depth coding illustrates the organizational hierarchy of precapillary arterioles, the capillary bed, and the postcapillary venule. The inset binary image shows the orientation of capillaries (green) parallel to the long axis of the myocyte (red). The myocyte was filled with sulforhodamine through microinjection. **(B)** The capillary bed displays varying lengths of capillary segments, with shorter segments indicated by red arrows (<10 μm) and longer segments by a white arrow (>100 μm). **(C)** A histogram and the distribution curve showing the distribution of different capillary segment lengths (495 capillary segments from 18 mice). **(D)** A correlation scatterplot and linear fit (*R* ^2,^ ∼0.018) of capillary diameter and segment length indicates weak relationship (Pearson’s R, −0.132) between the two. **(E)** Examples demonstrate capillary local narrowing (red arrow) and the location of pericytes (magenta). **(F)** A zoom-in of the arrow-pointed area in, **(E,G)** Summary data showing the capillary diameter of control (white arrow in **(F)** 4.41 ± 0.27 μm) and local narrowing (red arrow in **(F)** 2.20 ± 0.12 μm) area (14 capillaries from 6 independent preparations). ***p < 0.05 with T-test.

We tracked the septal artery to examine the arterioles and capillaries. However, only the superficial arteries and arterioles and their offshoots were able to be examined (Figure 2) using monophoton confocal or spinning disk confocal imaging. The daughter branches were observed using a Z-stack of images with 0.75–1 μm intervals. The capillary orders are not easy to track due to the depth and the density of the packed arrangement. Importantly, the organizational hierarchy of capillaries in heart differs significantly from that in the brain and retina (Longden et al., 2023; Mughal et al., 2023; Grant et al., 2019), where capillaries are classified according to their order in a sequential branching pattern. Instead, the capillaries in the heart form a dense and simpler mesh-like network. This network is characterized by extensive branching and anastomoses, with longer capillary segments oriented parallel to the long axis of the cardiac myocytes. Therefore, it is easy to identify and distinguish capillaries from precapillary arterioles, but it is difficult to distinguish between mother and daughter branches in the capillary bed.

### Capillaries

As shown in Figures 2A,B, the orientation and size of the precapillary arterioles are distinctly different from those of the capillaries. Since almost all the capillaries align tightly and longitudinally with the long axis of the cardiac myocytes immediately after their branching from the arterioles, we speculate that the diameters of all the capillaries are similar regardless of the segment length. Indeed, the capillary diameter measures 4.96 ± 0.1 μm under perfusion pressures ranging from 40 to 60 mmHg in the presence of BDM (30 mM). This number is smaller than the corrected value from the earlier work on fixed tissues (Bassingthwaighte et al., 1974; Kassab and Fung, 1994). The averaged capillary segment length was measured to be 58.7 ± 3.8 μm (495 capillary segments, 18 mice. Figure 2C), with the shortest segment measuring less than 10 μm, and the longest exceeding 180 μm (Figures 2B–D), which is considerably shorter than what was observed in rat heart and skeletal muscle (Fraser et al., 2012; Feldstein et al., 1978), but similar to pig heart capillaries (Kassab and Fung, 1994). At the present time these length differences might be functionally important. Figure 2D demonstrates that the correlation between capillary segment length and capillary diameter is weak (Pearson’s correlation coefficient = −0.132), yet statistically significant. The weak linear regression (*R* ^2^ ∼0.018), with a slope of approximately −0.005, indicates that the capillary diameter decreases by 0.1 μm for every 20 μm increase in segment length. The total capillaries occupy approximately 13.5% ± 0.8% of the total tissue volume (31 images, 21 mice).

### Capillary diameter dynamics

Capillary local narrowing is sometimes observed in our preparation, especially in the absence of BDM (Figures 2E–G). The diameter of the narrowest area in pressurized vascular systems is ∼2 μm (2.20 ± 0.12 μm, 14 capillaries from 6 independent preparations, Figure 2G). The distance of the narrow center to the pericyte soma is 26.3 ± 4.3 μm, suggesting that pericyte cell soma is not directly involved in all capillary local narrowing.

#### Structural diversity of cardiac pericytes

##### Intimacy: pericytes and myocytes

**FIGURE 3:**
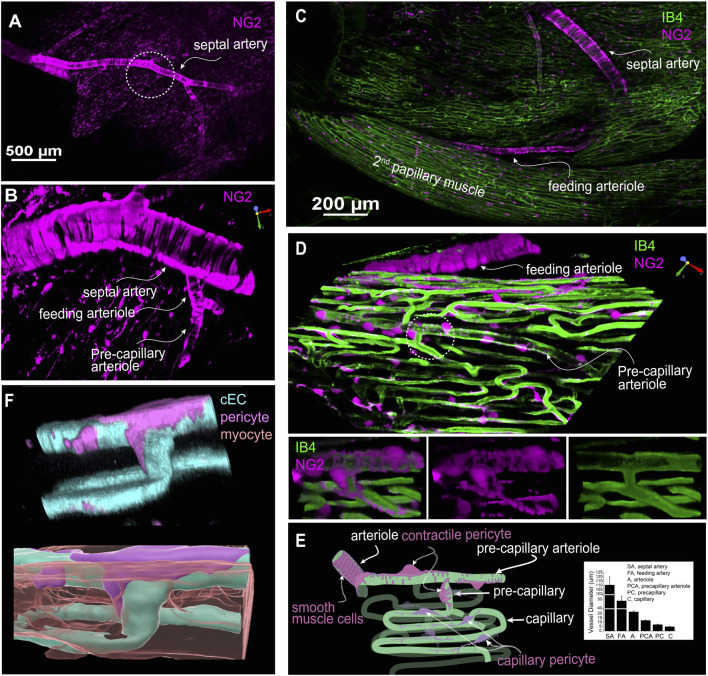
The geometry and structure of the microvascular bed of the mouse right ventricle papillary muscle. **(A)** A representative image of an NG2DsRed mouse heart showing that both vascular smooth muscle cells and pericytes are NG2 positive. **(B)** Zoomed-in region of the circled area in **(A)** showcasing the septal artery, feeding arteriole, and precapillary arteriole through imaging with NG2DsRed. **(C)** A representative image showing the branch of septal artery and feeding arteriole to the second papillary muscle. **(D)** Magnified 3D image of a region of the second papillary muscle showing a high-resolution image of the feeding arteriole, precapillary arterial and capillaries. Lower panel: zoomed-in image of the circled area from main panel. Note that the precapillary arteriole is sparsely covered by the contractile pericytes (often called ensheathing pericytes). **(E)** A cartoon drawing that depicts the microvessels and pericytes in papillary microcirculation. Ventricular myocytes fill the “apparently empty” space between the capillaries. Inlet shows the diameter of different segment of the vessel bed at 40–80 mmHg (n = 8 to 29 vessels, from 7 to 19 mice). **(F)** A pseudo-color 3D rendered image showing a pericyte, capillaries, and a myocyte (lower panel). The myocyte(s) is translucent to show its location with respect to the pericytes and cECs. **(A–D)** are from living Z-Prep in the presence of BDM; **(F)** is taken from a fixed heart tissue. The cEC, pericyte, and myocyte are revealed by IB4, NG2 and mitotracker, respectively.

**FIGURE 4:**
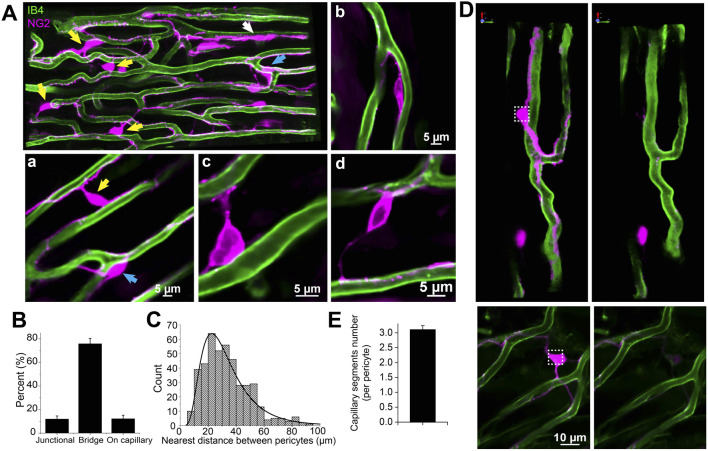
The subtypes and density of capillary pericyte in mouse right ventricle papillary muscle (Z-Prep). **(A)** NG2DsRed (A, Aa, Ab) and PDGFRβ (Ac, Ad) mouse heart image displaying pericytes (magenta) and microvessels (green). Various types of pericytes are indicated by arrows: yellow for bridging pericytes located between capillaries, blue for junctional pericytes located at bifurcation area, and white for pericytes located directly on capillaries. Zoomed-in images (Aa to Ad) provide detailed views of pericyte localization. Aa depicts both bridging and junctional pericytes, while Ab shows a pericyte directly attached to a capillary. Ac and Ad highlight bridging pericytes. Pericytes in Ac to Ad are PDGFRβ (magenta) positive cells. **(B)** Summary data presenting the distribution of different pericyte subtypes (399 pericytes from 17 mice). **(C)** Histogram and the distribution curve showing the nearest distance between two pericytes (435 pericytes from 18 mice). **(D)** Images showing pericytes and their processes before (left panels) and after photo-bleaching (right panels). **(E)** Summary data showing the number of capillaries that one pericyte covers (n = 20 pericytes from 4 independent preparations).

**FIGURE 5:**
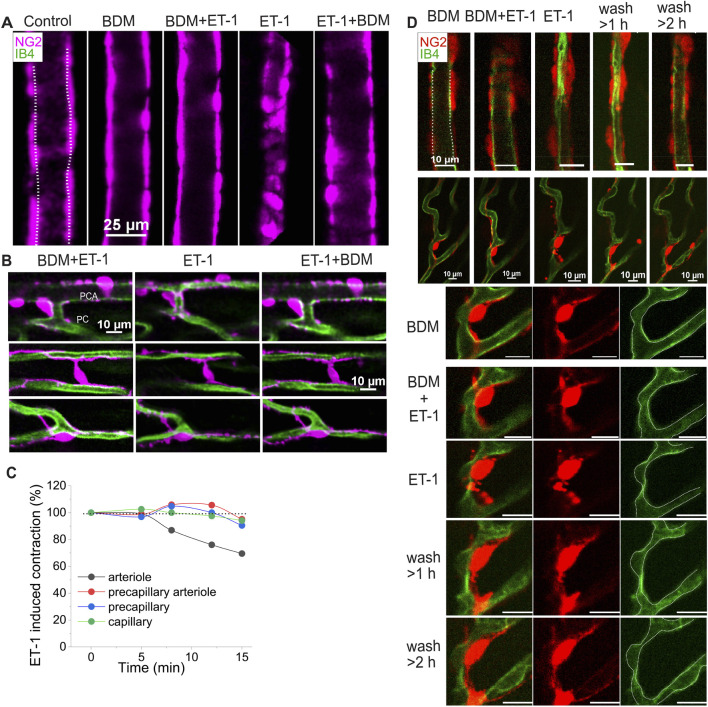
The dynamic change of cardiac pericytes and capillary to ET-1. **(A–C)** Representative images and data demonstrate the reversible response of different vessel types to ET-1 (10 nM, <30 min). **(A)** Images show the response of a feeding arteriole to ET-1. **(B)** Images illustrate diameter changes in a precapillary arteriole (top panel) and capillaries (middle and bottom panels) adorned with different pericyte types, in the presence (left and right) and absence (middle) of BDM on top of ET-1. These images were obtained from the same Z-Prep as in Figure 3C. **(C)** Time course of diameter changes in different vessel types in response to ET-1 (10 nM). **(D)** Example images show the reversible changes in arteriole and pericyte morphology, and the irreversible narrowing of capillaries following ET-1 (10 nM) treatment. After prolonged perfusion with ET-1 (10 nM, >30 min), arteriole diameter gradually recovered (top panel), whereas capillary diameter did not (middle and bottom panels). The bottom panel shows a zoomed-in view of a pericyte from middle panel. Notably, pericyte morphology was altered in the presence of ET-1 and restored after extensive perfusion with ET-1 free solution; however, the focal capillary narrowing remained. Dashed lines trace the vessel walls.

In this study, DsRed-tagged NG2 or tdTomato-tagged PDGFRβ transgenic mice were employed to identify pericytes (Figures 2E,F; Figures 3A–D; Figures 4A,D, 5). Despite the expression of both NG2 and PDGFRβ in arterial smooth muscle cells (ASMCs), these markers proved sufficient for effectively distinguishing pericytes from ASMCs using Z-Prep (Zhao et al., 2020a; Longden and Lederer, 2024; Longden et al., 2023; Zhao et al., 2020b; Hariharan et al., 2022; Berthiaume and Shih, 2019). As depicted in Figure 3 and supported by our prior research (Zhao et al., 2020a; Zhao et al., 2020b), pericytes were readily distinguishable from ASMCs based on their distinct location within the capillary beds and their characteristic cellular morphology (Zhao et al., 2020a; Longden et al., 2023; Zhao et al., 2020b). In the cardiac microenvironment, the compact and tight packing of myocytes results in shorter capillary segments between myocyte layers and many bifurcation angles closer to 90° (Figure 3F). Consequently, the spatial arrangement of pericyte somas and processes becomes particularly intriguing (Figures 3E,F). Even though it is challenging to identify the location and spread of each pericyte with respect to the other cells that surround it, it can be done. We used a simplification to estimate 3 types of pericyte locations each with a distinct character. 1. 12.0% ± 2.6% of the pericytes were identified as those located at the junctional areas where capillaries bifurcate (Figures 4Aa,B). 2. 12.2% ± 3.0% of the pericytes were located on the capillaries themselves. 3. 75.8% ± 4.5% of the pericytes formed a bridge between at least two capillaries. Examples of these three types are shown in Figures 4A,B. While all the pericytes are likely to have regions of exposure to ventricular myocytes, the “bridging pericytes” dominate in their exposure to and contact with ventricular myocytes! In addition, this is the predominant pericyte type in mouse heart. As the bridging pericytes stretch from one capillary to two or more capillaries (Figures 4D,E), much of the cell body and the connecting arms are surrounded closely by the sarcolemmal membranes of the ventricular myocytes. In many cases there are regions of intimate juxtaposition between such pericytes and their nearby myocytes. This spatial organization of the cardiac pericytes is thus very different from the capillary-centric localization pattern of pericytes observed in the retina and other parts of the brain (Mughal et al., 2023; Longden et al., 2021; Gonzales et al., 2020) and has not been reported before to the best of our knowledge.

##### The surprisingly high abundance of pericytes

We also measured the volume and the number of pericytes relative to capillary segment length and the number of cardiac myocytes. The total volume of the pericytes including processes occupies ∼4.8 ± 0.06% of the total tissue volume, or 1/3 of the total capillary volume. The quantification of pericytes was conducted based on the total volume of the imaged muscle. On average, there are approximately 15 pericytes per 10^6^ μm^3^ of papillary tissue, indicating one pericyte for every two to three cardiac myocytes! This calculation considers the mean volume of a single myocyte, which measures about 28,894 ± 3,875 µm (10 cardiac myocytes from 9 mice) (Longden and Lederer, 2024). Here there is an estimated density of approximately 35 myocytes per 10^6^ μm^3^ of papillary muscle tissue.

##### Location and “reach” of pericytes

Additionally, we determined the pericyte abundance along the capillary segments. On average, there is one pericyte every ∼150 µm along mouse capillaries. (This differs from previous reports on rat left ventricles which showed a greater abundance--one pericyte every 60 µm (O'Farrell et al., 2017)). Given that the average capillary segment length in mouse is 58.7 ± 3.8 μm (Figure 2), it follows that each pericyte and its processes could reach up to three capillary segments but this “reach” depends on the local geometry. Note that this estimated “reach” of a pericyte was confirmed through photo bleaching experiments (Figures 4D,E). Moreover, we observed that the nearest distance between two pericyte somas is on average 32.5 ± 1.4 μm (n = 115 pairs of pericytes), suggesting that each pericyte spans across at least two capillaries, since the minimum distance between adjacent capillaries is approximately 20 μm (Bassingthwaighte et al., 1974; Feldstein et al., 1978), equivalent to the thickness of a single cardiac myocyte (Li et al., 2020).

#### Pericyte contraction and microvascular blood flow regulation

**FIGURE 6:**
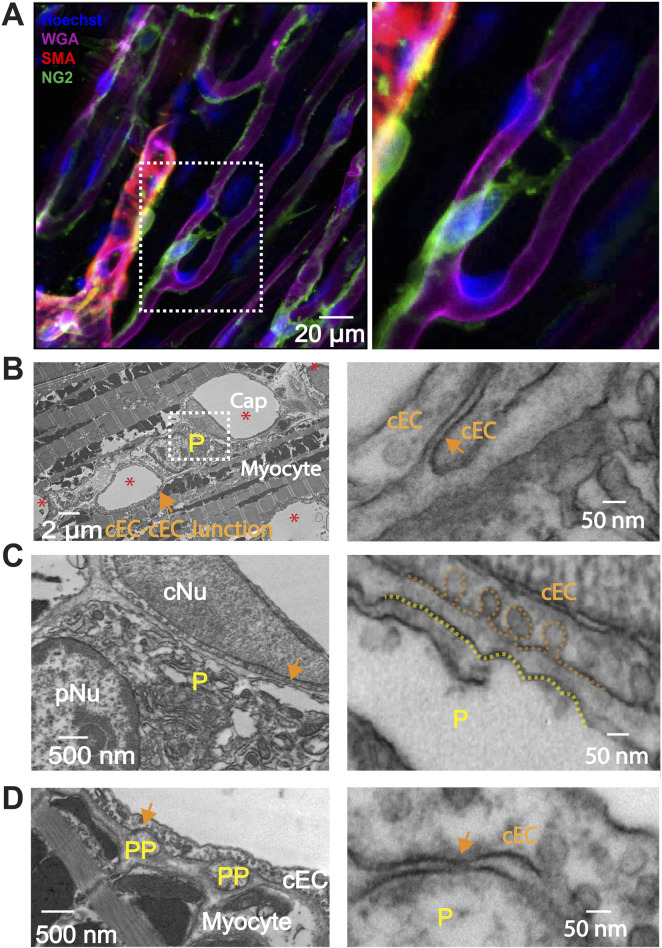
Immunostaining of smooth muscle actin (SMA) and Transmission Electron Microscopy (TEM) myograph of connections between pericyte, cEC and cardiac myocyte. **(A)** Whole-mount immunostaining reveals that smooth muscle actin (SMA, red) is expressed in pre-capillary arteriolar smooth muscle cells but not in pericytes (green). The papillary muscle was stained using anti-NG2 (green), anti-SMA (red), WGA (magenta), and Hoechst to identify capillary pericytes, smooth muscle cells, endothelial cells, and nuclei, respectively. Right panel, zoomed-in image of the boxed area in the left panel. **(B–D)** Transmission Electron Microscopy (TEM) myographs illustrating the structural interactions among pericytes, myocytes and capillary endothelial cells (cEC) in the mouse heart. **(B)** Cross section of mouse papillary muscle showing five capillaries (Cap, *) and one pericyte soma (P). cNu, capillary endothelial cell nucleus; pNu, pericyte nucleus. Cap, capillary. **(C)** Magnification of the framed area in **(B)**. **(D)** Cross section highlighting two pericyte processes (PP). The right panels of **(B–D)** magnify the corresponding arrowed areas showcasing junctional structures and space between cEC membranes, between the pericyte soma and cEC membranes, and between pericyte process membranes and cEC membranes.

That some types of pericytes contract and thereby narrow the diameter of capillaries to regulate blood flow is established in diverse tissues including brain (O'Farrell et al., 2017; Korte et al., 2024; Sancho et al., 2024; Hartmann et al., 2021). To examine the responsiveness of capillaries and the mechanisms under which the capillary diameters change, we applied endothelin-1 (ET-1) (e.g., 5 or 10 nM) in the vascular lumen. This application led to significant decrease in capillary diameter and other vascular elements. In our Z-prep experiments it is important to minimize motion of the Z-prep. In many experiments we have used BDM to reduce or block movement produced by contracting ventricular myocytes. As shown in Figure 5A, ET-1 (10 nM) did not change the arteriole diameter in the presence of BDM (30 mM). When BDM was washed away, the diameters of both arterioles and capillaries were reduced. The changes of the vessels were restored after addition of BDM (Figures 5A,B). Time course analysis demonstrates that the arteriole diameter change precedes the capillaries (Figure 5C). More interestingly, for the precapillary arteriole, the diameter reduction is likely attributable to the processes (Figure 5B, upper panel), whose constriction almost closes the arteriole. For the capillaries, ET-1 induces the cell body of bridging and junctional pericytes to shorten and round-up (Figure 5B, middle and bottom panels). In the presence of ET-1, the processes of all the pericytes developed a “granulated” or “lumpy-bumpy” appearance (Figures 5B,D). The mechanisms that underlie this morphological transformation and the diameter change remain unknown, as alpha smooth muscle actin (SMA) was barely detected in capillary pericytes (Figure 6A), consistent with the observations in other tissues including brain (Gonzales et al., 2020; Hartmann et al., 2021; Vanlandewijck et al., 2018; Nehls and Drenckhahn, 1991). When the vessels were perfused longer (>1 h) with ET-1 (10 nM) (Figure 5D), even extensive perfusion with ET-1 free solution cannot reverse the change of capillaries, whereas the arteriole diameter recovered (Figure 5D, top panel) and the pericytes were morphologically restored (Figure 5D, bottom panel).

#### Intercellular connections in heart

Enhanced specific communications between pericytes and capillary endothelial cells are reported in the brain at macroscopic sites called “peg and socket” connections. However, the exact nature and abundance of the enhanced communication is suggested by the presence of gap junctions in pericytes and endothelial cells and rare couplings consistent with clusters of gap junctions (Hari et al., 2020). There does not appear to be evidence of specific electrical synapses or of one or a cluster of gap junctions. For us to investigate the physical and signaling relationship between cEC’s and pericytes in heart, we used the tools of transmission electron microscopy. This was done using the same mouse right ventricle papillary muscle preparation as the Z-prep. Figures 6B–D show our findings from this investigation. First we did not find evidence of “peg-and-socket” connections in contrast to reports for brain microvessels (Ornelas et al., 2021; Abdelazim et al., 2022; Allsopp and Gamble, 1979; Bonney et al., 2022). There was evidence that the neighboring cells (pericytes and cEC’s) had “intimate” regions where both cells were as close to each other as was seen in peg-and-socket connections. Among the 67 pericyte cross sections (including processes or soma) in 19 capillary segments (2 independent preparations), 14 displayed regions that could have contained gap junctional structures (Figure 6D), where the closest distance between the capillary endothelial cell (cEC) membrane and the membrane of pericyte processes was less than 20 nm. However, the closest distance between the pericyte soma and the cEC membrane was approximately 40 nm (Figure 6C), a distance too large to support a gap junctional structure. So, like the data from brain and related peg-and-socket systems, perhaps it is the pericyte processes where pericytes communicate with cEC’s. It is also possible that we missed the gap junction due to limited sampling.

## Discussion

Pericytes are small pleomorphic cells associated primarily with capillaries and appear to have a distinct set of roles in regulating blood flow in heart and in other tissue (Longden et al., 2023; Hariharan et al., 2022; Gonzales et al., 2020; Hartmann et al., 2021; Hall et al., 2014; Hamilton et al., 2010). They are attached to the outside of the capillary endothelial cells that form the capillaries--the small tube-like structures of the smallest diameter element of the vascular system. This location places them on the exterior of the capillary wall which makes them “mural” cells analogous to the vascular smooth muscle cells that surround the somewhat larger arterioles and end-arterioles that connect the blood flow supply to the capillaries themselves. Published evidence identifies multiple morphologically distinct pericytes types in the brain that are associated with specific branches of the capillary tree (Grant et al., 2019; Hariharan et al., 2022; Gonzales et al., 2020; Hashitani et al., 2018; Fernandez-Klett et al., 2010). In brain, published findings in brain suggest electrical connections between pericytes and cEC’s through a structural entity called a “peg and socket” junction (Hari et al., 2020; Albargothy et al., 2023). However, the molecular connection between the different cell-types remains to be established. Both cell-types have a membrane potential of about −35 mV (Zhao et al., 2020a; Sancho et al., 2022). Here we have presented images of cardiac pericytes that look like pericytes in other tissues with respect to their organization on the cEC’s. There are two wide-spread similarities. A typical pericyte frequently appears to be a “bump on a log” nuclear protrusion with cytoplasmic thin strands that travel along part of the length of the capillary (tens of microns) (Higuchi et al., 2000; Zimmermann, 1923) (Figure 3). In brain and heart there are NG2 positive pericyte structures that may sit at the bifurcation of a small arteriole that splits into two capillaries (Longden et al., 2023; Gonzales et al., 2020). These structures appear to function as a directionally controlling pre-capillary sphincter (Gonzales et al., 2020). However, there are also some important differences. Specifically, the cardiac pericytes send extensions directly to nearby capillaries and also to more distant capillaries with these longer extensions traveling on the surface of ventricular myocytes or between them. We have also identified the appearance of pre-capillary “sphincters” in heart that appear to be a transition cell type resembling both single smooth muscle cells and also pericytes. They may entwine bifurcating arterioles as they transition to two outbound capillaries (Figure 3D). While there is evidence that the capillary pericytes can contract (Hartmann et al., 2021; Dalkara et al., 2024), there is insufficient data at the present time to characterize the full nature of the control of this and their kinetics in heart.

The imaging of pericytes in heart shown here raises challenging questions several of which are discussed below. First, how does the observed distinctive anatomy of cardiac pericytes suggest they work in the hypothesized electro-metabolic signaling (EMS) of heart? (Zhao et al., 2020a; Longden and Lederer, 2024; Longden et al., 2023). What experiments can be done to test these ideas? What can we learn about pericytes from their contractile behavior? What does the diversity of pericyte length and shape suggest as pericyte extensions traverse heart tissue?

### Microcirculatory structure in heart

Following arteries and arterioles, where the pattern of smooth muscle cells is clear, fewer smooth muscle cells are seen in the branching vessels. Instead, the vessels are covered with pericytes (Figure 3). While both smooth muscle cells and pericytes are NG2-positive, smooth muscle cells are short, ring-shaped and densely packed, which are easily identified and distinguished from pericytes, especially *in situ* (Figures 3, 4). Some researchers refer to all the vessels that are adorned with pericytes as capillaries, because pericytes were initially defined as cells that decorate capillaries (Mughal et al., 2023; Zimmermann, 1923; Cai et al., 2023; Zambach et al., 2021; Cai et al., 2018). Others identify the vessels with “ensheathing” pericytes as proximal capillaries (Hariharan et al., 2022; Hashitani et al., 2018) or pre-capillary arteriole because these pericytes are smooth muscle actin-positive and function similarly to smooth muscle cells (Grant et al., 2019; Gonzales et al., 2020; Fernandez-Klett et al., 2010). Our observations support the latter concept and suggest that the vessels covered with contractile pericytes may be pre-capillary arterioles (Figure 3). Accordingly, the pericytes on the pre-capillaries are referred as “contractile pericytes” or “pre-capillary pericytes”. A specific single contractile pericyte that is located on a short pre-capillary segment is thereby referred as pre-capillary “sphincter”. Unlike what has been observed in the brain and other tissues where capillary arrangements are cascaded in a sequence, in the heart, capillaries are distributed relatively regularly and largely at a single “level” (Figures 2–4) in “working” myocardium. These capillaries are primarily oriented parallel to the long axis of the cardiac myocytes and are inter-connected with short perpendicular stretches (see Figure 2), like the arrangement found in skeletal muscle (Jacobsen et al., 2022; Emerson and Segal, 1997; Plyley et al., 1976). Thus, small diameter capillaries run parallel to the myocytes with branches at approximately right angles. We do not recommend renaming the cardiac capillaries with order numbers as done by Kassab and his group (Kassab et al., 1993; Kassab and Fung, 1994; Kassab, 2005). Re-stating the capillary organization that we see in heart--there is a single capillary type serving functioning heart muscle but with two orientations. In brain, the complexity is much greater. The cardiac capillaries have been morphologically divided into subtypes based on their three locations: pre- and post-capillary and working capillary with appropriate pericytes for each type (Higuchi et al., 2000). Our recent work, however, reveals that there are three main functional subtypes of pericytes associated with the working capillaries. The first type runs along the length of the capillary. The second, termed a “bridging pericyte,” originates on one capillary, extends closely alongside a neighboring cardiomyocyte with intimate contact, and terminates on a distinct parallel capillary. The third type is located at capillary junctions. Interestingly, despite these differences in location and pericyte association, capillaries exhibit similar diameters, regardless of segment length or pericyte coverage (Table 1).

**TABLE 1:** Comparison of capillary and pericyte in brain and heart.

Feature	Brain/Retina	Heart (current work)
Capillary network	Hierarchically organized, branching with clear order (1st, 2nd …) (Mughal et al., 2023; Hartmann et al., 2022)	Dense, mesh-like. Oriented parallel to cardiac myocyte with short connecting capillary segments
Capillary segment length	∼35–∼70 μm (Longden et al., 2021; Cudmore et al., 2017)	Average ∼60 μm. Ranges from <10 μm to >180 μm
Capillary diameter	∼3.0–15 μm (Gonzales et al., 2020; Hartmann et al., 2022; Cudmore et al., 2017; Peppiatt et al., 2006; Swe et al., 2018; Reeson et al., 2018). Focal narrowing (∼1–5 μm) (Reeson et al., 2018)	Average ∼4.96 μm at 40–60 mmHg perfusion pressure. Focal narrowing (∼2 μm)
Pericyte type	Ensheathing (*locate on 1*st *to 4*th *order capillary*, SMA+) (Mughal et al., 2023; Gonzales et al., 2020)	Contractile pericytes (*locate on precapillary arteriole and precapillary*, SMA+). Investigations continue
	Mesh (transitional zone, most SMA+) (Mughal et al., 2023; Gonzales et al., 2020)	
	Thin-strand (*on true capillary, SMA-*) (Mughal et al., 2023; Gonzales et al., 2020) en passant ∼77% and junctional ∼23% (Mughal et al., 2023; Gonzales et al., 2020)	Capillary pericytes: (SMA-. *Junctional ∼12%, bridging ∼76% and on capillary (en passant) ∼12%, SMA-)*
Pericyte density	1.1 to 1.4 pericytes per 100 μm of capillary segment (varies by depth) (Cudmore et al., 2017)	One per ∼150 μm of capillary length
Pericyte-capillary contact	Mostly capillary-bound with short lateral processes	Long projections, span between capillaries

### Cardiac pericytes morphology, classification and blood flow regulation

While the contractile properties of pericytes have been studied extensively in some tissues, such as in the brain, research on the fundamental functions of pericytes in the heart is still lacking. Most studies have focused on changes in pericytes under pathological conditions such as heart failure and myocardial ischemia. One reason for this is the constant beating of the heart, which makes it challenging to observe real-time changes in pericytes. That is precisely what we need to study in heart. Now, thanks to our new research model, we can monitor the dynamic changes of the capillary diameter and pericyte morphology in active tissue. Our imaging of live heart tissue clearly shows the variation in the capillary diameter and the relationship between the diameter and the surrounding pericytes. For example, the capillary diameter can be altered in pathologic situations such as ischemia (O'Farrell et al., 2017; Peppiatt et al., 2006). Capillary closure may be observed in heart, brain and other tissues (O'Farrell et al., 2017; Li et al., 2022; Freitas and Attwell, 2022) and this can decrease or even stop blood flow - when it occurs. Capillary dilation was also observed in the living tissues other than heart, especially in the presence of vasodilators or when the tissue was exposed in acute hypoxia (Hariharan et al., 2022; Longden et al., 2021; Peppiatt et al., 2006; Ernst and Bek, 2023). Nevertheless, it is still unclear how pericytes regulate the capillary diameter under physiological and pathological conditions.

In our study, we found that local narrowing of capillaries occurs more often in the absence of a vasodilator. In addition, the narrow areas do not necessarily coincide with the locations of pericyte somas. While our results on this topic might differ from the findings of Attwell’s group and others (O'Farrell et al., 2017), their data come from tissues that were either fixed or without perfusion (O'Farrell et al., 2017; Hall et al., 2014). Many studies suggested that pericyte contraction reduces capillary flow and decreases capillary diameter. On the other hand, damage or loss of pericytes may lead to the stoppage or insufficiency of local blood flow (O'Farrell et al., 2017; Hall et al., 2014; Freitas and Attwell, 2022; Greif and Eichmann, 2014; Kaul et al., 2023). Our observation *in situ* heart tissue strongly suggest that the reduction of capillary diameter is secondary to the constriction of their upstream arterioles. For example, when supplying perfusates that contain ET-1, arterioles are the first to response and constrict (Figure 5). This is consistent with the report in the brain (Fernandez-Klett et al., 2010). Later studies demonstrate that longer and stronger depolarizing stimulation would enable capillary pericytes to contract (Fernandez-Klett et al., 2010), suggesting that capillary pericytes are not necessarily involved in capillary blood flow regulation as contractile agents under physiological conditions (Schwarting et al., 2023). The diameter change starts a while after the arteriole constricted, suggesting that either the arteriolar smooth muscle or precapillary pericytes are more sensitive to ET-1 or other vasoconstrictors. Indeed, the circular processes are rich with contractile filaments (Figure 6A) (Gonzales et al., 2020; Hashitani et al., 2018). Another possibility is that the delayed response to ET-1 stimulation results from ET-1 reaching the capillaries later than the upstream arterioles, which requires further investigation. Channelrhodopsin excitation contracts the brain pericytes and reduce the blood flow (Nelson et al., 2020). At present, the literature is largely in agreement that all the pericytes are contractile regardless of their location, although the pericytes along the brain capillary contract at a much slower rate, exerting a more gradual effect on blood flow regulation (Hartmann et al., 2021). However, some observations made on capillary pericytes (en passant pericyte, thin strand pericyte) do not support this conclusion. Most of the measurements were conducted on the “capillaries” with ensheathing or mesh pericytes which are smooth muscle actin positive (Gonzales et al., 2020; Hall et al., 2014; Li et al., 2022). The diameter of these “capillaries” is generally >6 µm (Cai et al., 2018; Li et al., 2022; Freitas and Attwell, 2022; Baez et al., 1977), which fall into our precapillary arteriole category (Grant et al., 2019; Hall et al., 2014). We therefore refer to the pericytes on the pre-capillary arteriole as “contractile pericytes” to acknowledge their properties, functions, and location. This classification of pericyte form and placement reflects a sophisticated level of vascular regulation, hinting at a complex system where each pericyte subtype may be finely tuned to meet the specific demands of the tissue it serves.

To state this clearly, the mechanisms by which pericytes regulate capillary diameter and microcirculation remains unclear, despite the apparent roles of pericytes in ischemic conditions in the heart (O'Farrell et al., 2017). As depicted in Figure 6A and supported by some previous research, capillary pericytes do not express measurable smooth muscle actin (SMA), the primary filament protein responsible for constriction in smooth muscle cells and contractile pericytes (Gonzales et al., 2020; Hartmann et al., 2021; Vanlandewijck et al., 2018). However, the application of ET-1 did induce a decrease in capillary diameter as well as the morphological change of the pericyte processes, as shown in Figure 5. Thus, we have reason to believe that the decrease in capillary diameter might be due to the structural and/or functional change of pericytes. However, surprisingly, after washing out ET-1, we found that the narrowing of capillary remains, while the structure of pericytes and their processes seemed restored (Figure 5D). These data might suggest that 1) capillary endothelial cells were damaged permanently; or 2) the change of pericyte morphology and capillary diameter are two independent “ischemic reactions”. As shown in Figures 5B,D, the precapillary arterioles constrict completely which might result in the “stopping of flow” in the downstream capillaries (Sweeney and Sarelius, 1989).

### Pre-capillary sphincters, smooth muscle cells and pericytes

Originating in 1937 when Zweifach and his colleagues described the precapillary sphincter in the mesentery of frogs (BW, 1937), this particular structure has long been thought to be universally present in the microvessels of all tissues and has been included in physiology textbooks. However, this structure is typically depicted through artistic drawings in textbooks rather than from original experimental observations. Traditionally described as a ring of smooth muscle at the arteriolar-capillary junction, sphincters are indicated to regulate blood flow into the capillaries. Even though the structure and function of the precapillary sphincter were hot topics and had been widely studied in the last century (Baez et al., 1977; Nicoll, 1971; Folkow et al., 1984; Berman et al., 1972; Siggins and Weitsen, 1971), the precapillary sphincter was not well-defined due to technical limitations. Firstly, three-dimensional imaging was unavailable, making it difficult to accurately illustrate the sphincter’s morphology. Secondly, vessels in *ex vivo* or *in vitro* preparations were not perfused, so their diameters did not reflect physiological conditions. Lastly, there were no biomarkers to define the cell types. Although these technical limitations have now been resolved, the definition, territory, structure, and function of the precapillary sphincters remain unclear, especially in the heart. Recent research indicates that the precapillary sphincter in brain regulates local blood flow (Zambach et al., 2021), and its loss or impaired dynamics might cause neurovascular dysfunction (Zambach et al., 2021) (Cai et al., 2023)^,^ (Nikolakopoulou et al., 2019)^,^ (Winkler et al., 2014). Among all smooth muscle cells or pericytes capable of constriction, it exhibits the greatest contraction upon stimulation (Zambach et al., 2021). In skeletal muscle, where the demand for oxygen can fluctuate dramatically, these sphincters are also postulated to play a critical role in directing blood flow in response to local metabolic needs. However, the existence and functional role of precapillary sphincters in the heart have not been clear. In fact the existence and the function of sphincters has been a topic of debate among vascular biologists for many years (Sakai and Hosoyamada, 2013). Some researchers even question whether this structure is indeed present in tissues other than the mesentery and have even pointed out that it is not universally present (Sakai and Hosoyamada, 2013; Poole and Edward, 2019). In this study, we have identified sphincter-like structures in the heart, as shown in Figure 3. These structures consist of one or a few pericytes located between capillaries and arterioles. While some scholars refer these pericytes as “mesh pericytes” (Longden et al., 2023; Mughal et al., 2023; Grant et al., 2019; Hariharan et al., 2022), we prefer to name them “contractile pericytes” or “precapillary pericytes” (Longden et al., 2023). They are among the last group of pericytes that express smooth muscle actin with high contractile activity. Even though it is still unclear how this type of pericyte regulates blood flow into the downstream capillaries, the ET-1-induced constriction (Figure 4) of this pericyte was observed and the presence of α-smooth muscle actin was confirmed (Longden et al., 2023). Given that the classical concept of the precapillary sphincter may not apply uniformly across all tissues, the fundamental principle of blood flow regulation at the microvascular junction is a critical aspect of tissue physiology that may be carried out by various mechanisms across different organ systems.

### Limitation of the work

Although the *ex vivo* Z-Prep model was designed to mimic physiological conditions by controlling perfusion pressure and temperature, it remains imperfect and non-physiological. For example, there are neither blood cells nor blood proteins in the flowing fluid within the vasculature. Additionally, the use of motion inhibitors such as BDM or TTX can themselves affect the function of both cardiomyocytes and the vascular bed. Dye loading may stimulate vascular responses, potentially influencing the data output. Despite these limitations, our high-resolution live imaging approach offers a novel perspective for visualizing and interpreting physiological and pathological phenomena and may help advance the understanding of cardiac microcirculation.

In summary, here we have presented a new view on how pericytes are organized in the mammalian heart. Using a perfused physiological model of mouse heart, this study has described a new geometry and fine structure of the microvasculature with a focus on pericytes. Three pericyte identities appear to exist in heart: 1. Pre-capillary sphincter pericytes; 2. Longitudinal pericytes; and 3. Bridging pericytes. This report thus lays the foundation for future functional studies and provides guidance for identifying mural cell types on and around the capillaries in heart. Additionally, our real-time imaging enables dynamic tracking of capillary diameter changes under stress and challenges in heart. Finally, this work provides the organizational foundation for future studies on the physiological roles of cardiac pericytes and some basic guidance on how they may work.

## Data Availability

The original contributions presented in the study are included in the article/supplementary material, further inquiries can be directed to the corresponding author.
